# Evaluating the efficacy of endotracheal and intranasal epinephrine administration in severely asphyxic bradycardic newborn lambs: a randomised preclinical study

**DOI:** 10.1136/archdischild-2024-327348

**Published:** 2024-09-04

**Authors:** Justine de Jager, Romy Pothof, Kelly J Crossley, Georg M Schmölzer, Arjan B te Pas, Robert Galinsky, Nhi T Tran, Nils Thomas Songstad, Claus Klingenberg, Stuart B Hooper, Graeme R Polglase, Calum T Roberts

**Affiliations:** 1 Division of Neonatology, Department of Pediatrics, Leiden University Medical Center, Leiden, The Netherlands; 2 Department of Obstetrics and Gynaecology, Leiden University Medical Center, Leiden, The Netherlands; 3 The Ritchie Centre at Hudson Institute of Medical Research, Clayton, Victoria, Australia; 4 Centre for the Studies of Asphyxia and Resuscitation, University of Alberta, Royal Alexandra Hospital, Edmonton, Alberta, Canada; 5 Department of Obstetrics and Gynaecology, Monash University, Melbourne, Victoria, Australia; 6 Research Group Child and Adolescent Health, Faculty of Health Sciences, UiT The Arctic University of Norway, Tromsø, Norway; 7 Department of Pediatrics and Adolescence Medicine, University Hospital of North Norway, Tromsø, Norway; 8 Department of Paediatrics, Monash University, Melbourne, Victoria, Australia; 9 Monash Newborn at Monash Children's Hospital, Clayton, Victoria, Australia

**Keywords:** Resuscitation, Neonatology, Physiology

## Abstract

**Objective:**

Intravenous epinephrine administration is preferred during neonatal resuscitation, but may not always be rapidly administered due to lack of equipment or trained staff. We aimed to compare the time to return of spontaneous circulation (ROSC) and post-ROSC haemodynamics between intravenous, endotracheal (ET) and intranasal (IN) epinephrine in severely asphyxic, bradycardic newborn lambs.

**Methods:**

After instrumentation, severe asphyxia (heart rate <60 bpm, blood pressure ~10 mm Hg) was induced by clamping the cord in near-term lambs. Resuscitation was initiated with ventilation followed by chest compressions. Lambs were randomly assigned to receive intravenous (0.02 mg/kg), ET (0.1 mg/kg) or IN (0.1 mg/kg) epinephrine. If ROSC was not achieved after three allocated treatment doses, rescue intravenous epinephrine was administered. After ROSC, lambs were ventilated for 60 min.

**Results:**

ROSC in response to allocated treatment occurred in 8/8 (100%) intravenous lambs, 4/7 (57%) ET lambs and 5/7 (71%) IN lambs. Mean (SD) time to ROSC was 173 (32) seconds in the intravenous group, 360 (211) seconds in the ET group and 401 (175) seconds in the IN group (p<0.05 intravenous vs IN). Blood pressure and cerebral oxygen delivery were highest in the intravenous group immediately post-ROSC (p<0.05), whereas the ET group sustained the highest blood pressure over the 60-min observation (p<0.05).

**Conclusion:**

Our study supports neonatal resuscitation guidelines, highlighting intravenous administration as the most effective route for epinephrine. ET and IN epinephrine should only be considered when intravenous access is delayed or not feasible.

**WHAT IS ALREADY KNOWN ON THIS TOPIC**

- Endotracheal and intranasal epinephrine can be administered more rapidly and easily than intravenous epinephrine during neonatal resuscitation, but are largely ineffective in asystolic newborns.

**WHAT THIS STUDY ADDS**

- Endotracheal and intranasal epinephrine were less effective compared with intravenous epinephrine in terms of success rates and time to restore cardiac function in bradycardic newborn lambs.
- Intranasal epinephrine achieved a return of spontaneous circulation at a similar rate and time interval as endotracheal epinephrine, and may be a more convenient and faster administration route when intravenous epinephrine is delayed or not feasible.

**HOW THIS STUDY MIGHT AFFECT RESEARCH, PRACTICE OR POLICY**

- Future studies should focus on optimising epinephrine administration techniques, taking into account differences between asystolic and bradycardic newborns.
- Use of intranasal epinephrine, as a quicker and more easily administered alternative to established methods, has the potential for evaluation in clinical trials.

## Introduction

 Perinatal asphyxia, a prolonged lack of oxygen in infants around the time of birth, is one of the leading causes of neonatal death,^1^ with approximately 580 000 infants dying annually.^2^ A large proportion of these deaths may be prevented by effective cardiopulmonary resuscitation (CPR), including positive pressure ventilation, chest compressions (CC) and epinephrine administration.^3^,^5^ Although less than 0.1% of infants at birth require epinephrine,^6 7^ these infants are at high risk of major adverse outcomes, such as death and neurological disabilities.^8^

Neonatal resuscitation guidelines recommend intravenous epinephrine administration via an umbilical venous catheter (UVC).^3^,^5^ However, placing the UVC can be difficult and may not be feasible due to a lack of equipment or expertise, particularly in resource-limited settings.^9^ As alternatives, guidelines recommend epinephrine administration via an intraosseous needle or endotracheal tube (ET).^3^,^5^ The ET route may be faster compared with intravenous epinephrine, particularly as the ET tube is usually inserted early during CPR for ventilation purposes.^10^ Intranasal (IN) epinephrine via an atomiser spray has also been proposed as no invasive procedures are required.^11^ However, previous studies suggest that both ET and IN epinephrine are less effective in achieving return of spontaneous circulation (ROSC) compared with intravenous epinephrine in asystolic newborns.^611^,^14^ Notably, increasing the dose of ET epinephrine improved the time to and the rates of ROSC to similar levels as intravenous epinephrine. However, the higher ET doses resulted in a prolonged and greater overshoot in blood pressure following resuscitation, which was associated with an increased incidence of cerebral microbleeds.^14^

Previous preclinical studies have investigated the utility of other routes of epinephrine administration in asystolic newborns, where there is no cardiac output present.^1113^,^15^ However, most newborns requiring CPR are not asystolic.^16^ It is unclear whether ET and IN epinephrine are efficacious in neonates with less severe asphyxia. Therefore, in this study, we aimed to determine the efficacy of ET and IN epinephrine in restoring cardiac function in severely asphyxic newborn lambs with low but ongoing cardiac output. We hypothesised that ET and IN epinephrine would be less effective than intravenous epinephrine in restoring cardiac function, measured as the time to achieve ROSC, in severely asphyxic, bradycardic newborn lambs.

## Methods

The online supplemental methodology file describes the instrumentation, resuscitation and statistical methods in detail, as described previously, and in keeping with published guidelines.^14 17^

Immediately prior to surgery, lambs were randomly allocated, using a web-based random sequence generator (www.random.org/lists), to one of three treatment groups:

1. ‘IV Epinephrine’ (n=8), treated with 0.02 mg/kg of intravenous epinephrine (0.1 mg/mL) according to standard neonatal resuscitation guidelines, followed by 0.9% saline flush (5 mL).
2. ‘ET Epinephrine’ (n=8), treated with 0.1 mg/kg of endotracheal epinephrine (1 mg/mL), followed by a few seconds of sustained positive pressure.
3. ‘IN Epinephrine’ (n=8), treated with 0.1 mg/kg of intranasal epinephrine (1 mg/mL) in one nostril using an Intranasal Mucosal Atomization Device (LMA MAD Nasal, Teleflex, Morrisville, North Carolina, USA), after suctioning of the respective nostril.

Blinding of the resuscitation team was not possible due to the route of administration of epinephrine. After inducing asphyxia, rather than continuing to asystole, resuscitation commenced when the mean blood pressure had decreased to ~10 mm Hg and the heart rate was below 60 bpm. Resuscitation commenced with ventilation in air. After 1 min, the fraction of inspired oxygen was increased to 1.0, and CCs were initiated. At 2 min, epinephrine was administered and repeated every 3 min thereafter until cardiac function was restored. We defined this as ROSC, which was indicated by diastolic blood pressure >20 mm Hg and spontaneous heart rate >100 bpm and increasing, as determined by the researcher leading the resuscitation. If ROSC was not achieved after three allocated treatment doses, two ‘rescue’ doses of standard-dose intravenous epinephrine could be administered. CPR was ceased if ROSC was not achieved by 15 min. Lambs that achieved ROSC were ventilated for 60 min. Ventilation settings were manually adjusted to target SaO_2_ (arterial oxygen saturation) 90–95% and PaCO_2_ (arterial partial pressure of carbon dioxide) 35–45 mm Hg, as determined by periodic arterial blood gas measurements. Thereafter, the lambs were euthanised. The primary outcome was time to ROSC. We previously demonstrated a mean (±SD) time of 186 (±33) seconds in our intravenous epinephrine group.^14^ To demonstrate a 30% change in time to ROSC assuming a power of 80% and an alpha value of 0.05, six animals per group were required. We planned, a priori, to include eight animals per group to optimise the availability of post-ROSC physiological data for analysis, assuming reduced survival in some treatment groups as evident from our previous study.^14^

## Results

24 lambs were instrumented in this study. Two lambs were excluded from the analysis: one lamb achieved ROSC through ventilation alone (ET group) and one lamb was growth restricted (IN group).

### Lamb characteristics

Lamb characteristics prior to initiation of the study were similar between all treatment groups (table 1).

**Table 1:** Lamb characteristics

	Intravenous epinephrine (n=8)	ET epinephrine (n=7)	IN epinephrine (n=7)
Gestational age (days)*	140±1.2	140±1.0	140±1.0
Birth weight (kg)*	4.6±0.5	4.7±0.4	4.7±0.4
Gender (male)†	4 (50)	4 (57)	3 (43)
Fetal characteristics: after instrumentation and prior to asphyxia			
pH*	7.249±0.04	7.245±0.05	7.264±0.04
PaO_2_ (mm Hg)*	20.0±6.2	17.2±3.3	18.4±4.2
PaCO_2_ (mm Hg)*	64.6±7.9	66.9±5.0	66.3±6.6
SaO_2_ (%)*	50.0±19.3	38.7±12.7	46.6±14.7
SctO_2_ (%)*	47.9±2.7	48.6±8.8	47.2±9.3
Lactate (mmol/L)*	3.3±0.7	3.4±0.9	2.7±0.6
End asphyxia characteristics: after asphyxia immediately before resuscitation			
pH*	6.951±0.04	6.935±0.03	6.930±0.04
PaO_2_ (mm Hg)*	0.6±1.2	0.6±0.6	0.3±0.5
PaCO_2_ (mm Hg)*	107.4±15.0	112.6±7.8	117.3±10.1
SaO_2_ (%)*	2.4±1.9	1.8±0.3	1.6±0.3
SctO_2_ (%)*	32.6±14.4	28.5±11.2	29.6±7.0
Lactate (mmol/L)*	8.8±1.4	9.3±1.6	9.0±0.8
Postmortem organ weights			
Lung weight (g)*	155.2±34.0	163.8±29.8	170.8±19.9
Brain weight (g)*	54.2±2.7	58.1±4.0	58.2±3.7

* Values are mean±SD.

† n (%).

ET, endotracheal; IN, intranasal; PaCO_2_, arterial partial pressure of carbon dioxide; PaO_2_, arterial partial pressure of oxygen; SaO_2_, arterial oxygen saturation; SctO_2_, cerebral tissue oxygen saturation.

### Response to treatment and survival

The response to treatment and survival of the lambs are presented in table 2.

**Table 2:** Response to treatment and survival

	Intravenous epinephrine (n=8)	ET epinephrine (n=7)	IN epinephrine (n=7)
ROSC			
With allocated treatment alone*	8/8	4/7	5/7
In response to rescue intravenous epinephrine*	N/A	1/3	1/2
Total*	8/8	5/7	6/7
Allocated treatment doses:			
One dose*	8/8	3/7	3/7
Two doses*	0/8	1/7	2/7
Three doses*	0/8	3/7	2/7
Rescue intravenous epinephrine doses:			
One dose*	0/8	1/7	1/7
Two doses*	0/8	2/7	1/7
Time to ROSC (s)†‡	173±32	360±211	401±175§
Survival to end study*¶	8/8	5/7	6/7

* Values are n/N.

† Mean±SD.

‡ Measured from initiation of ventilation.

§ p<0.05 intravenous versus IN epinephrine.

¶ Assessed at 60 min after ROSC.

ET, endotracheal tube; IN, intranasal; ROSC, return of spontaneous circulation.

**Figure 1:**
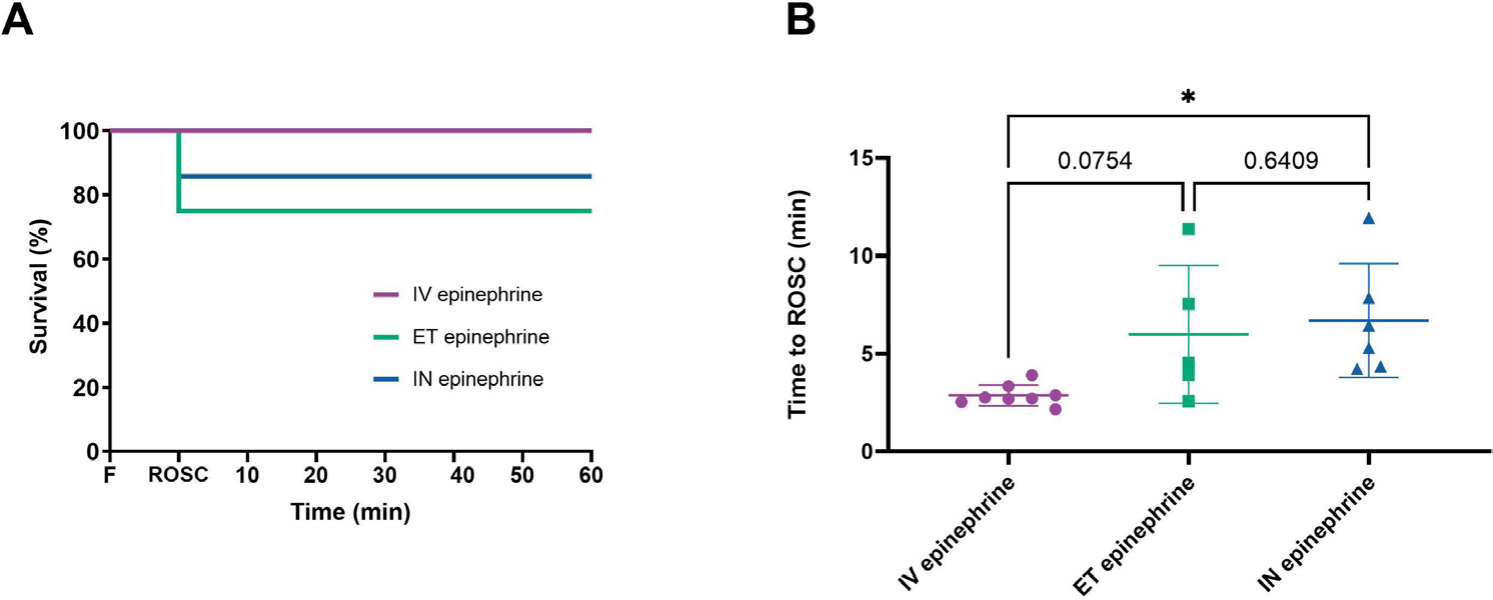
Outcomes and survival. (**A**) Survival throughout the study in lambs administered intravenous (n=8), ET (n=7) and IN epinephrine (n=7). (**B**) Time to ROSC (mean±SD). Time to ROSC was measured from the onset of ventilation. Data are shown for the lambs that achieved ROSC: intravenous epinephrine (●, n=8), ET epinephrine (■, n=5) and IN epinephrine (▲, n=6). * indicates p<0.05. ET, endotracheal; F, fetal; IV, intravenous; IN, intranasal; ROSC, return of spontaneous circulation.

The proportion of lambs to achieve ROSC in response to allocated treatment was not different between the intravenous (8/8, 100%), ET (4/7, 57%) and IN epinephrine groups (5/7, 71%). Including rescue intravenous epinephrine, ROSC occurred in 5/7 (71%) of the ET lambs and 6/7 (86%) of the IN lambs (figure 1). The time to achieve ROSC in those lambs that did was significantly longer in the IN group compared with the intravenous group (figure 1).

### Physiological response to CPR

**Figure 2:**
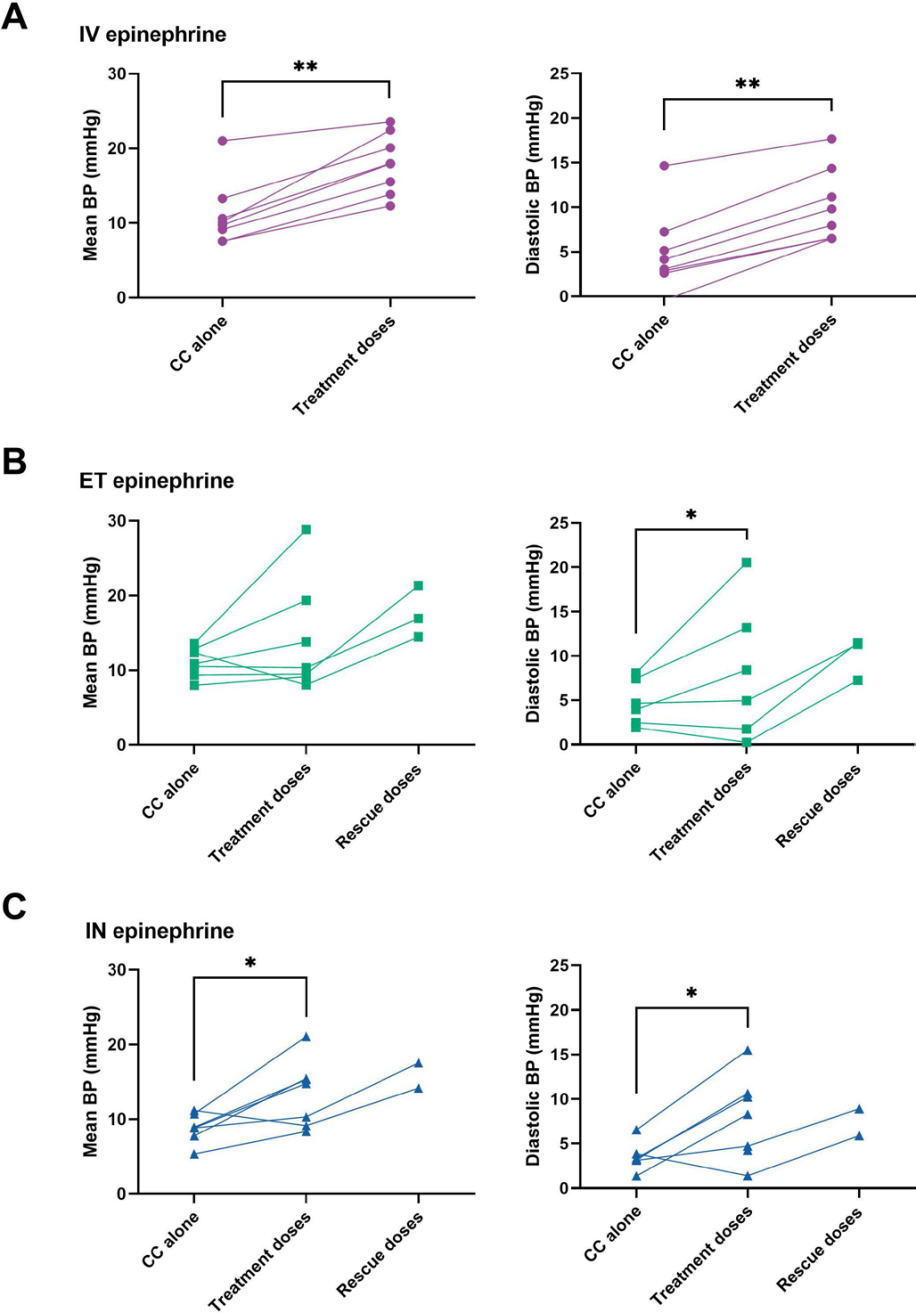
Individual lamb changes to mean and diastolic blood pressure during CPR. Mean BP and diastolic BP of individual lambs during CC alone, during treatment with intravenous, ET or IN epinephrine (CC+1–3 doses of allocated treatment), and during treatment with rescue intravenous epinephrine (CC+3 doses of allocated treatment+1–2 doses of intravenous epinephrine) in the (**A**) intravenous epinephrine (●), (**B**) ET epinephrine (■) and (**C**) IN epinephrine (▲) groups. Each data point is the mean per lamb over the respective time period. The time periods analysed vary between individual lambs, depending on the duration of CPR. * indicates p<0.05, ** indicates p<0.01. CPR, cardiopulmonary resuscitation; CC, chest compressions; diastolic BP, diastolic blood pressure; ET, endotracheal; IN, intranasal; IV, intravenous; mean BP, mean blood pressure.

Individual changes to mean and diastolic blood pressure are presented in figure 2, while physiological parameters during CPR are shown in online supplemental figure 1. There were no differences in physiological parameters during CPR between the treatments. Within all groups, mean pulmonary blood flow significantly increased after intravenous, ET and IN epinephrine, respectively. Mean carotid blood flow significantly increased in response to epinephrine in the intravenous group. Diastolic blood pressure significantly increased in all groups after intravenous, ET and IN epinephrine, respectively. Mean blood pressure increased in the intravenous group after intravenous epinephrine, and in the IN group after IN epinephrine. Systolic blood pressure significantly increased in response to epinephrine only in the intravenous epinephrine group.

### Physiological responses following ROSC

Physiological parameters after ROSC are shown in online supplemental figure 2. During the first 10 min after ROSC, mean pulmonary blood flow was significantly higher in the intravenous group compared with the IN group. Mean carotid blood flow and mean, systolic and diastolic blood pressure were significantly higher in the intravenous group compared with the ET and IN group in the first minutes after ROSC. The time for mean blood pressure to peak was significantly shorter in the intravenous group compared with the IN group (online supplemental figure 3). Heart rate was significantly higher in the intravenous group compared with ET between 80 and 160 s after ROSC.

From 15–60 min after ROSC, mean, systolic and diastolic blood pressures were significantly higher in the ET group compared with the intravenous and IN groups. Relative to the IN group, mean carotid and pulmonary blood flow were significantly higher at 15 min after ROSC in the intravenous and ET groups, respectively. No differences in heart rate were observed between the groups over the 15–60 min after ROSC.

### Blood gas and oxygenation after ROSC

Blood gases and cerebral oxygen kinetics are presented in online supplemental figure 4. Fraction of inspired oxygen was not different between groups throughout the study (data not shown). At ROSC, arterial partial pressure of oxygen (PaO_2_) and arterial oxygen saturation (SaO_2_, blood gas) were significantly higher in the intravenous group compared with the ET and IN group. At 3 min post-ROSC, PaO_2_ was significantly higher in the IN group than in the intravenous group. Cerebral oxygen delivery in the intravenous group was significantly higher compared with the ET and IN group at 6 min and from ROSC to 6 min thereafter, respectively. The cerebral oxygen extraction was significantly higher in the IN group compared with the intravenous group at ROSC. No other significant differences were observed. Peripheral oxygen saturation readings were excluded from the analysis due to unreliability.

### Plasma epinephrine concentrations

**Figure 3:**
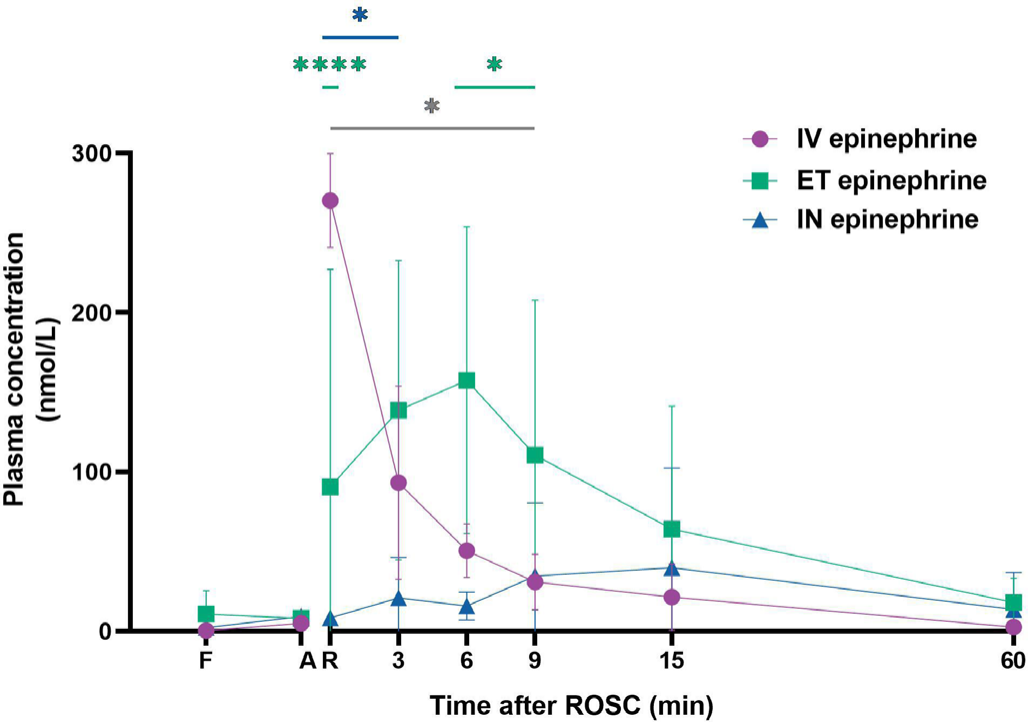
Plasma epinephrine concentrations Plasma epinephrine concentrations at fetal control (F), after asphyxia immediately before resuscitation (A), at ROSC (R) and for 60 min after ROSC. Data are shown for the lambs that achieved ROSC: intravenous epinephrine (●, n=8), ET epinephrine (■, n=5) and IN epinephrine (▲, n=6). The time scale of the first 15 min after ROSC has been magnified to aid visualisation. Values are mean±SD. * indicates p<0.05, **** indicates p<0.0001. Green (*) indicates statistical significance between intravenous and ET; blue (*) indicates statistical significance between intravenous and IN; grey (*) indicates statistical significance between ET and IN. ET, endotracheal; IV, intravenous; IN, intranasal; ROSC, return of spontaneous circulation.

Plasma epinephrine concentrations in lambs achieving ROSC are shown in figure 3. At ROSC, plasma epinephrine levels were 270±29 nmol/L for the intravenous group, 90±137 nmol/L for ET and 8±4 nmol/L for IN. Post-ROSC, intravenous epinephrine concentrations rapidly decreased to near fetal levels at 6 min. ET epinephrine plasma levels peaked at 157±96 nmol/L at 6 min and remained significantly higher than intravenous and IN epinephrine until 15 min after ROSC, then returned to near fetal levels. IN epinephrine concentrations gradually increased to 40±63 nmol/L at 15 min, but remained similar to fetal levels throughout the experiment.

## Discussion

We found that intravenous epinephrine is the most efficacious administration route, compared with ET and IN epinephrine, to achieve ROSC in severely asphyxic, bradycardic newborn lambs. We also demonstrated that ET and IN administered epinephrine performed similarly in terms of achievement of ROSC, time to ROSC and physiological response in the immediate post-ROSC phase.

Intravenous epinephrine administration resulted in the highest rates of ROSC and within the shortest time. The suboptimal responses to ET and IN epinephrine are likely due to reduced bioavailability associated with airway and nasal absorption. In the transitioning neonate, residual lung liquid, low pulmonary blood flow and the relatively thick respiratory epithelium at birth may further complicate absorption.^9 18 19^ This corroborates with the finding that intravenous epinephrine increased mean, systolic and diastolic blood pressure more prominently and consistently during CPR compared with ET and IN.

At ROSC, plasma epinephrine concentration following IN administration was ~1/30th of intravenous and ~1/8th of ET levels. Despite this, 5/7 (71%) lambs in the IN group achieved ROSC after the allocated treatment. Similarly, a previous study in lambs showed comparable timing and rates of ROSC despite different plasma epinephrine levels after ET epinephrine, suggesting other factors influence ROSC.^20^ This brings into question whether the intravenous epinephrine plasma concentration of 270 nmol/L at ROSC may be redundant in bradycardic lambs, or even harmful. Exogenous epinephrine can aggravate the rebound hypertension following ROSC, leading to microbleeds^21^,^23^ and cerebral hyperoxygenation.^24^,^27^ Indeed, in our study, intravenous epinephrine demonstrated the greatest and most rapid overshoot in carotid blood pressure and oxygen delivery following ROSC compared with ET and IN epinephrine in the immediate post-ROSC phase.

Following the immediate post-ROSC phase, plasma epinephrine levels gradually increased in the ET and IN group, indicating continued systemic absorption of epinephrine after ROSC. This finding is consistent with the sustained higher blood pressures in the ET group compared with the intravenous and IN group over the 60-min observation. It is possible that the lung liquid functions as a barrier for ET epinephrine to reach the pulmonary epithelium and vasculature, resulting in delayed, sustained absorption of ET epinephrine. Excessive exposure to epinephrine is associated with haemodynamic instability and increased mortality.^28^

Our findings of reduced efficacy and impaired recovery with ET epinephrine align with existing neonatal recommendations favouring intravenous administration over ET epinephrine.^3^,^5^ While IN epinephrine has not been acknowledged in current guidelines, its effects were similar to ET, consistent with the study of Songstad *et al* in asystolic lambs.^11^ Importantly, IN epinephrine is non-invasive and can be administered more quickly than ET, making it a potentially more suitable temporary alternative when intravenous administration is delayed or not feasible. Previous canine CPR studies showed that IN epinephrine reaches the systemic circulation and effectively increases coronary perfusion pressure.^29 30^ In the neonatal intensive care unit, IN administration is effective and easy for analgosedation, especially during urgent procedures without intravenous access.^31^ Future studies are needed to evaluate the applicability of IN epinephrine during neonatal resuscitation.

As another alternative when intravenous access is not feasible, recent neonatal resuscitation guidelines recommend using intraosseous (IO) epinephrine administration.^3^,^5^ Data on IO access use in neonates are largely from case reports, highlighting complications.^32^ However, a recent nationwide German study showed that IO access was feasible and safe in most neonates.^33^ Furthermore, simulation studies demonstrated that IO access is quicker than intravenous access,^34^,^36^ and a preclinical study in newborn lambs found intravenous and IO equally effective regarding ROSC and physiological responses after ROSC.^15^ Given the apparent efficacy of the IO route, the ET and IN routes would only be advantageous if they could be used substantially more quickly, as a temporising measure. Indeed, a recent study demonstrated increased rates of ROSC in infants receiving initial ET epinephrine compared with initial intravenous epinephrine supporting its role when intravenous access is delayed, although 40% of infants receiving ET required subsequent intravenous rescue.^37^

When evaluating routes of epinephrine administration during neonatal resuscitation, it may be relevant to differentiate between asystolic and bradycardic infants, especially as excessive use of epinephrine has been shown to be associated with detrimental side effects.^28^ Kumar *et al* previously demonstrated that asystolic infants require more extensive resuscitation compared with bradycardic infants.^16^ In this study, the rates of ROSC were higher, and the time to ROSC shorter, in ET and IN lambs than in previous preclinical studies with similar treatment protocols in asystolic lambs.^11 14^ However, this study did not directly compare bradycardic lambs to asystolic lambs, and future studies would be beneficial.

Animal losses and exclusions, along with variability in ROSC rates, reduced the study’s sample size and statistical power. The exclusion of the lamb that only received ventilation (ET) and the growth-restricted lamb (IN) was decided on a posteriori. Achievement of ROSC with ventilation alone was not anticipated, as previous studies in this model demonstrated that ROSC was only achieved after epinephrine administration, with 0/5 and 1/6 lambs in the saline control groups achieving ROSC without it.^11 14^ In clinical practice, most infants require only respiratory support, with CCs and medications being rare (0.1%).^7^ Although the excluded lamb’s response may have been physiological, it was excluded due to the study’s aim to compare different epinephrine administration routes. The growth-restricted lamb was excluded as these lambs have different cardiovascular haemodynamic responses to asphyxia compared with appropriately grown lambs.^38^ Future studies are needed to examine how epinephrine administration affects growth-restricted lambs specifically.

Moreover, despite similar size, anatomical differences between lambs and infants limit clinical extrapolation. Additionally, lung liquid drainage before asphyxia and anaesthesia are limitations of the preclinical design. The study investigated a single mode of asphyxia induction (acute umbilical cord occlusion), potentially restricting generalisability to other clinical scenarios. However, this study used a well-established preclinical model, specifically designed to investigate transition complicated by severe asphyxia. Another strength of the study includes randomisation of the three treatment groups.

## Conclusions

Consistent with current neonatal resuscitation guidelines, intravenous epinephrine is the most efficacious administration route compared with ET and IN epinephrine to restore cardiac function in severely asphyxic, bradycardic newborn lambs. Our findings only indicate that the use of ET or IN epinephrine may be appropriate when intravenous access is delayed or not feasible. Due to its low invasiveness and rapid delivery, IN may have potential in resource-limited settings.

## Supplementary material

10.1136/archdischild-2024-327348 online supplemental file 1

10.1136/archdischild-2024-327348 online supplemental file 2

## Data Availability

Data are available upon reasonable request.
