# Supplementary material for: Evaluating the efficacy of endotracheal and intranasal epinephrine administration in severely asphyxic bradycardic newborn lambs: a randomised preclinical study
Source: Arch Dis Child Fetal Neonatal Ed. 2024 Sep 4;110(2):e327348. doi: 10.1136/archdischild-2024-327348 (PMC12013545; doi:10.1136/archdischild-2024-327348)
Supplement: online supplemental file 1 [file fetalneonatal-110-2-s001.pdf]

# SUPPLEMENTARY METHODOLOGY FILE

## Instrumentation and delivery

Anaesthesia was induced in pregnant Border-Leicester ewes (*Ovis Aries*) at 140±1 days gestation (mean±SD; term ~148 days) with intravenous thiopentone sodium (20 mg/kg) and maintained with isofluorane (1.5-5%) in air/oxygen after intubation of the ewe. $FiO_2$ was adjusted to maintain maternal peripheral oxygen saturations of >95%. An intravenous cannula was inserted into a maternal jugular vein for infusion of 0.9% saline throughout the procedure.

The fetus was exteriorised via a laparotomy and immediately intubated with a 4.5mm cuffed endotracheal tube, and the endotracheal tube was clamped to prevent lung liquid loss during surgery. Flow probes (Transonic Systems, Ithaca, NY, USA), size 3 and 4 mm, were surgically placed around the left carotid artery and left main pulmonary artery, respectively. Heparinised saline-filled catheters were inserted into the right carotid artery (to measure blood pressure via a pressure transducer (PD10; DTX Plus Transducer, Becton Dickinson, Singapore)), left brachial artery (for arterial blood gas sampling) and right jugular vein (for venous blood gas sampling and analgesia infusion) as described previously (1). Peripheral oxygen saturation ($SpO_2$; Masimo, Radical 4, CA, USA) and cerebral tissue oxygen saturation ($SctO_2$; Casmed Foresight, CAS Medical Systems Inc, Branford, CT, USA) were measured. Temperature was measured by a rectal temperature probe to maintain an internal temperature of ~39°C. All physiological parameters were digitally recorded continuously at 1kHz a PowerLab A-D converter and stored using LabChart7 software (ADInstruments, Bella Vista, NSW, Australia). Immediately prior to delivery, lung liquid was passively drained. The umbilical cord was clamped to induce asphyxia, the lamb was weighed and transferred to an infant warmer (Fisher and Paykel Healthcare, Auckland, New Zealand). Respiratory support was withheld until mean blood pressure had decreased to ~10 mmHg and heart rate had fallen below 60 bpm.

**Resuscitation and Post-Resuscitation Care**

Resuscitation was initiated in air using positive pressure ventilation via a T-piece device (Neopuff; Fisher and Paykel Healthcare, Auckland, New Zealand) at 60 inflations per minute, with peak inflation pressure of 30 $cmH_2O$ and end-expiratory pressure of 5 $cmH_2O$. One minute after ventilation onset, chest compressions were initiated with a target of 90 compressions and 30 inflations per minute, and the fraction of inspired oxygen was increased to 1.00 as per resuscitation guidelines (2-4).

Two minutes after initiation of ventilation and every three minutes thereafter, epinephrine was administered via the allocated route. ROSC was defined as diastolic blood pressure >20 mmHg and spontaneous heart rate >100 bpm and increasing, as determined by the researcher leading the resuscitation. If ROSC was not achieved after three allocated treatment doses, two 'rescue' doses of standard dose IV epinephrine could be administered.

If ROSC was not achieved by 15 minutes, CPR was ceased. If ROSC was achieved, lambs were immediately transferred onto positive pressure-limited ventilation at 30/5 $cmH_2O$ (Dräger Babylog 8000+, Dräeger, Lübeck, Germany) for the first 10 minutes with heated humidified gas (F&P 950 System, Fisher and Paykel, Auckland, New Zealand). Thereafter, volume guarantee ventilation was commenced at tidal volume of 7 ml/kg until 60 minutes post-ROSC.

Plasma samples were collected from the brachial artery before cord clamping (fetal), at end asphyxia, and at ROSC, 3, 6, 9, 15 and 60 minutes after ROSC. Plasma epinephrine concentrations were determined by enzyme immunoassay according to manufacturer's instructions (Epinephrine 162 Research ELISA, catalogue #BA E-5100; LDN, Germany).

Blood gas samples (ABL30, Radiometer, Copenhagen, Denmark) were collected from the brachial artery and jugular vein before cord clamping (fetal), at end asphyxia, at ROSC, every 3 minutes after ROSC until 15 minutes, and then at 20, 25, 30, 40, 50 and 60 minutes. Cerebral oxygen kinetics

(oxygen delivery, $DO_2$; oxygen consumption, $VO_2$; cerebral oxygen extraction, COE) were calculated as described previously (5). Ventilation settings were adjusted to target $SaO_2$ 90-95% and $PaCO_2$ 35-45 mmHg, as determined by periodic arterial blood gas measurements. Lambs received analgesia from 10 minutes after ROSC to prevent any pain or distress and spontaneous breathing (Alfaxan 5-15 mg/kg/hr in 5% dextrose).

At the completion of the experiment or if the lambs failed to achieve ROSC, ewes and lambs were euthanized using an intravenous overdose of sodium pentobarbitone (100 mg/kg IV, Lethobarb, Virbac, Australia).

**Statistical analysis**

All lambs were analysed for the assessments made during CPR while only lambs achieving ROSC were included in the post-ROSC analysis. Data analysis was performed using GraphPad Prism v9 (GraphPad Software, CA, USA) and R Studio v4.1.17 (Posit Software, MA, USA). Baseline characteristics, time to ROSC, and time to peak mean blood pressure were compared using a one-way ANOVA. A Fisher's exact test was used to compare dichotomous outcomes. To assess physiological data, blood gases, cerebral oxygen kinetics, and plasma epinephrine concentration, a two-way repeated measures ANOVA or a mixed-effects model (depending on whether there was missing data) was used, with "group" and "time" as the factors and the Holm-Sidak *post hoc* multiple comparisons test to compare differences between and within groups. Post-ROSC physiological data were compared in time blocks of the first 10 minutes and last 45 minutes. Statistical significance was accepted at $p<0.05$. Data are reported as mean ± standard deviation unless otherwise stated.

## References


1. Polglase GR, Schmölzer GM, Roberts CT, *et al.* Cardiopulmonary Resuscitation of Asystolic Newborn Lambs Prior to Umbilical Cord Clamping; the Timing of Cord Clamping Matters! *Front Physiol*. 2020;**11**:902 doi: 10.3389/fphys.2020.00902 [published Online First: 20200730].
2. Wyckoff MH, Wyllie J, Aziz K, *et al.* Neonatal Life Support: 2020 International Consensus on Cardiopulmonary Resuscitation and Emergency Cardiovascular Care Science With Treatment Recommendations. *Circulation*. 2020;**142**:S185-s221 doi: 10.1161/cir.0000000000000895 [published Online First: 20201021].
3. Madar J, Roehr CC, Ainsworth S, *et al.* European Resuscitation Council Guidelines 2021: Newborn resuscitation and support of transition of infants at birth. *Resuscitation*. 2021;**161**:291-326 doi: 10.1016/j.resuscitation.2021.02.014 [published Online First: 20210324].
4. Aziz K, Lee HC, Escobedo MB, *et al.* Part 5: Neonatal Resuscitation: 2020 American Heart Association Guidelines for Cardiopulmonary Resuscitation and Emergency Cardiovascular Care. *Circulation*. 2020;**142**:S524-s50 doi: 10.1161/cir.0000000000000902 [published Online First: 20201021].
5. Andersen CC, Pillow JJ, Gill AW, *et al.* The cerebral critical oxygen threshold of ventilated preterm lambs and the influence of antenatal inflammation. *J Appl Physiol (1985)*. 2011;**111**:775-81 doi: 10.1152/japplphysiol.00214.2011 [published Online First: 20110630].
