# Supplementary material for: Evaluating the efficacy of endotracheal and intranasal epinephrine administration in severely asphyxic bradycardic newborn lambs: a randomised preclinical study
Source: Arch Dis Child Fetal Neonatal Ed. 2024 Sep 4;110(2):e327348. doi: 10.1136/archdischild-2024-327348 (PMC12013545; doi:10.1136/archdischild-2024-327348)
Supplement: online supplemental file 2 [file fetalneonatal-110-2-s002.pdf]

Supplementary Figure 1. Physiology during CPR

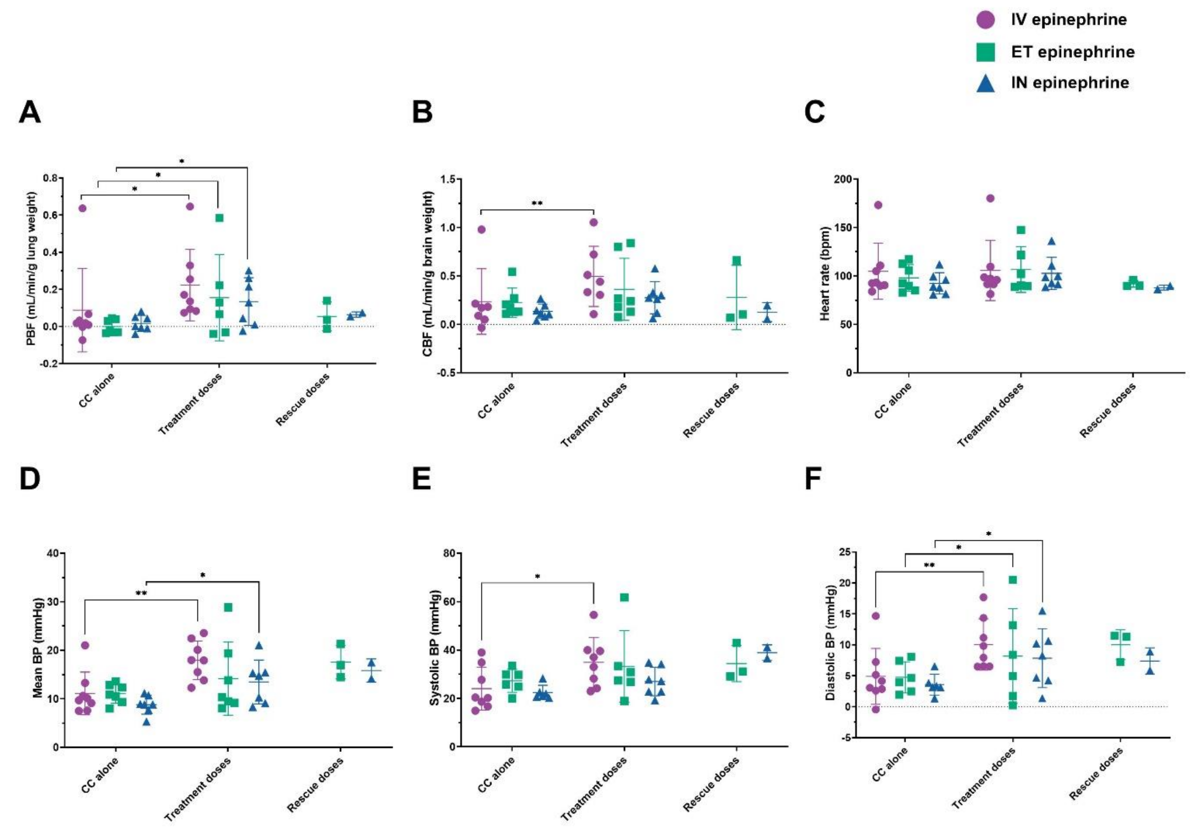

A) Mean pulmonary blood flow, B) mean carotid blood flow, C) mean heart rate, D) mean blood pressure, E) systolic blood pressure, and F) diastolic blood pressure during chest compressions (CC) alone, during treatment with IV, ET or IN epinephrine (CC + 1-3 doses of allocated treatment), and during treatment with rescue IV epinephrine (CC + 3 doses of allocated treatment + 1-2 doses of IV epinephrine) in the IV epinephrine (●), ET epinephrine (■), and IN epinephrine (▲) groups. *Each datapoint is the mean per lamb over the respective time period. Time periods analyzed vary between individual lambs, depending on the duration of CPR. Values are mean ± SD. * indicates p<0.05, ** indicates p<0.01. CPR = cardiopulmonary resuscitation; CC = chest compressions; PBF = pulmonary blood flow; CBF = carotid blood flow; Mean BP = mean blood pressure; Systolic BP = systolic blood pressure, Diastolic BP = diastolic blood pressure.*

Supplementary Figure 2. Physiology after ROSC

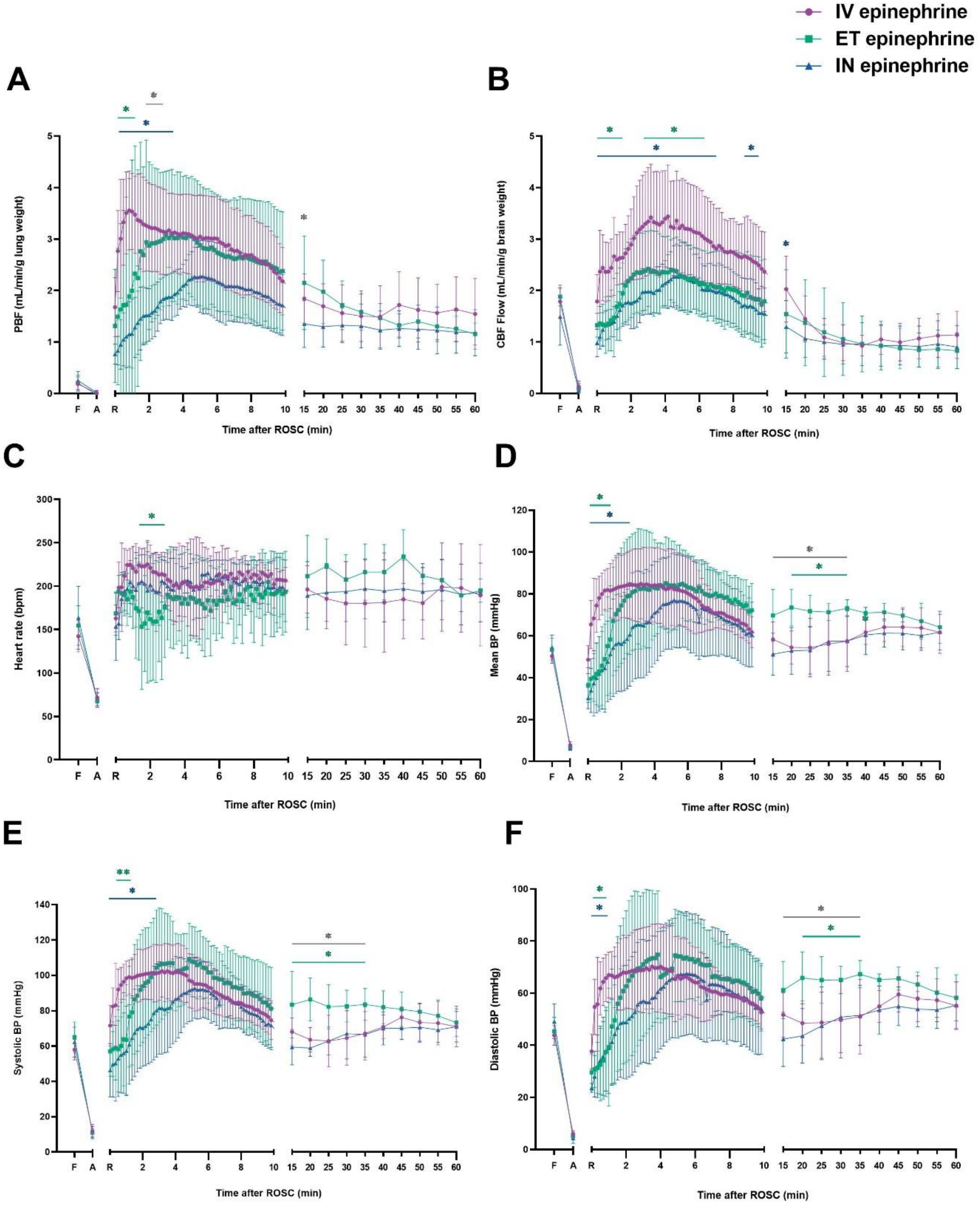

A) Mean pulmonary blood flow, B) mean carotid blood flow, C) mean heart rate, D) mean blood pressure, E) systolic blood pressure, and F) diastolic blood pressure, at fetal control (F), after asphyxia immediately before resuscitation (A), at ROSC (R), and for 60 minutes after ROSC. Data are shown for the lambs that achieved ROSC: IV epinephrine (●, n=8), ET epinephrine (■, n=5) and IN epinephrine (▲, n=6). *Values are mean ± SD.* indicates p<0.05, ** indicates p<0.01. Green (*) indicates statistical significance between IV and ET; Blue (*) indicates statistical significance between IV and IN. Gray (*) indicates statistical significance between ET and IN. ROSC = Return of Spontaneous Circulation; IV = intravenous; ET = endotracheal; IN = intranasal; PBF = pulmonary blood flow; CBF = carotid blood flow; Mean BP = mean blood pressure; Systolic BP = systolic blood pressure, Diastolic BP = diastolic blood pressure.*

Supplementary Figure 3. Time to peak mean blood pressure after ROSC

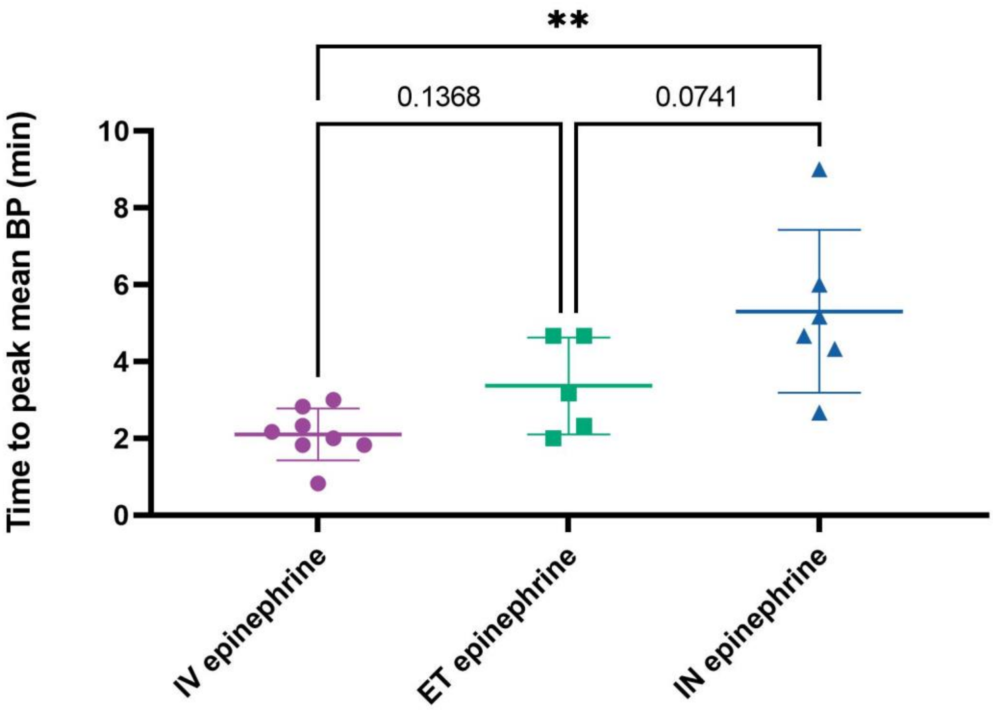

Data are shown for the lambs that achieved ROSC: IV epinephrine (●, n=8), ET epinephrine (■, n=5) and IN epinephrine (▲, n=6). *Values are mean ± SD. ** indicates p<0.01. IV = intravenous; ET = endotracheal; IN = intranasal; Mean BP = mean blood pressure; ROSC = Return of Spontaneous Circulation.*

Supplementary Figure 4. Blood gases and cerebral oxygen kinetics

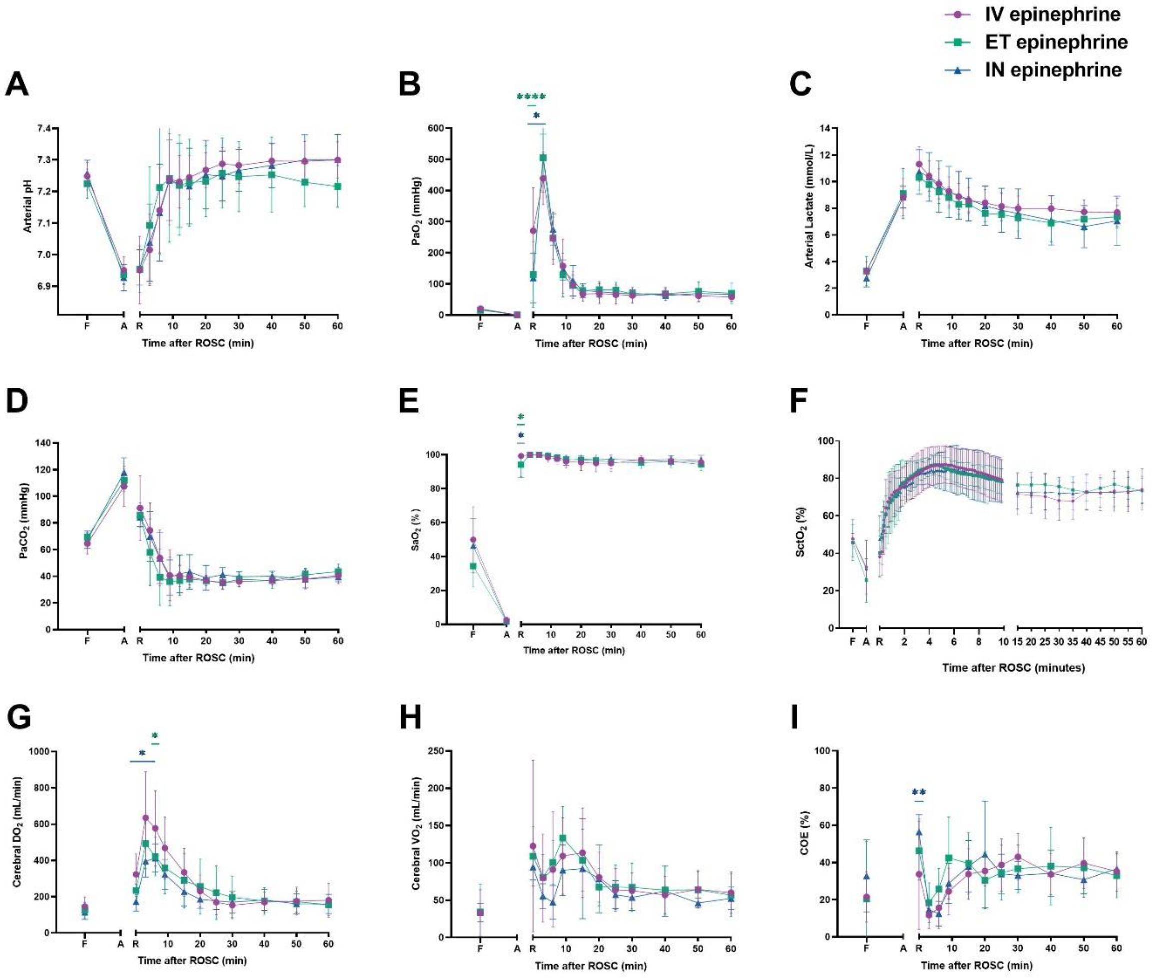

A) Arterial pH, B) arterial partial pressure of oxygen, C) arterial lactate, D) arterial partial pressure of carbon dioxide, E) arterial oxygen saturation, F) cerebral tissue oxygen saturation, G) cerebral oxygen delivery, H) cerebral oxygen consumption, and I) cerebral oxygen extraction at fetal control (F), after asphyxia immediately before resuscitation (A), at ROSC (R), and for 60 minutes after ROSC. Data are shown for the lambs that achieved ROSC: IV epinephrine (●, n=8), ET epinephrine (■, n=5) and IN epinephrine (▲, n=6). *Values are mean ± SD.* indicates $p<0.05$, ** indicates $p<0.01$, **** indicates $p<0.0001$. Green (*) indicates statistical significance between IV and ET; Blue (*) indicates statistical significance between IV and IN. ROSC = Return of Spontaneous Circulation; IV = intravenous; ET = endotracheal; IN = intranasal; PaO_2 = arterial partial pressure of oxygen; $PaCO_2$ = arterial partial pressure of carbon dioxide; SaO_2 = arterial oxygen saturation; $SctO_2$ = cerebral tissue oxygen saturation; DO_2 = oxygen delivery; VO_2 = oxygen consumption; COE = cerebral oxygen extraction.*
